# Damage-Induced Cell Regeneration in the Midgut of *Aedes albopictus* Mosquitoes

**DOI:** 10.1038/srep44594

**Published:** 2017-03-16

**Authors:** Maria Janeh, Dani Osman, Zakaria Kambris

**Affiliations:** 1 Biology Department, Faculty of Arts and Sciences, American University of Beirut, Beirut, Lebanon; 2 Faculty of Sciences III and Azm Center for Research in Biotechnology and its Applications, LBA3B, EDST, Lebanese University, 1300, Tripoli, Lebanon

## Abstract

Mosquito-transmitted diseases cause over one million deaths every year. A better characterization of the vector’s physiology and immunity should provide valuable knowledge for the elaboration of control strategies. Mosquitoes depend on their innate immunity to defend themselves against pathogens. These pathogens are acquired mainly through the oral route, which places the insects’ gut at the front line of the battle. Indeed, the epithelium of the mosquito gut plays important roles against invading pathogens acting as a physical barrier, activating local defenses and triggering the systemic immune response. Therefore, the gut is constantly confronted to stress and often suffers cellular damage. In this study, we show that dividing cells exist in the digestive tract of adult *A. albopictus* and that these cells proliferate in the midgut after bacterial or chemical damage. An increased transcription of signaling molecules that regulate the EGFR and JAK/STAT pathways was also observed, suggesting a possible involvement of these pathways in the regeneration of damaged guts. This work provides evidence for the presence of regenerative cells in the mosquito guts, and paves the way towards a molecular and cellular characterization of the processes required to maintain mosquito’s midgut homeostasis in both normal and infectious conditions.

Mosquitoes are well known vectors of human and animal diseases. The Asian tiger mosquito *Aedes albopictus* is an important vector for several pathogens, including Chikungunya, Dengue and the recently identified Zika virus^1^. This mosquito was identified in the Middle East 10 years ago and its population size has increased since then^2^. The presence of endogenous mosquito vectors together with climatic warming may lead to the spread of mosquito-borne diseases in the near future. It is therefore important to better understand mosquito immune response in order to develop effective control strategies against these vectors of diseases. Like all other insects, mosquitoes depend on innate immunity to fight pathogens^3^,^4^. Different immune responses have been described in mosquitoes including phagocytosis, melanization, complement-like mediated lysis and antimicrobial peptides (AMPs) production^5^,^6^,^7^. AMPs are released when pattern recognition receptors (PRRs) recognize pathogen associated molecular patterns (PAMPs) and trigger a downstream signal transduction cascade resulting in the activation of effectors’ responses^8^,^9^.

The gut constitutes an important component of the mosquito’s defense against foreign invaders. Besides its role in food digestion, the gut forms a physical barrier preventing dissemination of ingested pathogens. An example is the Anopheline mosquito species, where the midgut plays a role as a physical barrier against *Plasmodium* parasites due to the presence of the peritrophic matrix lining the midgut epithelium^10^. The mosquito gut epithelium is also able to clear microbes after the activation of local immune response. It has been shown that the Immune Deficiency (IMD) pathway is activated prior to the invasion of the midgut epithelium by the ookinetes in *Anopheles* mosquitoes^11^. Maintaining the integrity of mosquitoes’ gut is therefore indispensable for effective local immune defenses against harmful pathogens.

The alimentary canal of larval mosquitoes is nearly completely autolysed and replaced during pupation so that the adult digestive apparatus is largely built anew. A few studies have focused on the guts of mosquito larvae^12^, while curiously, the adult gut remains poorly explored. Food digestion, ingestion of cytotoxic compounds, enteric infections and molecules produced during the immune response are major gut stress-inducers^13^. In *Drosophila melanogaster*, the presence of such stress-inducers in the gut lumen result in cell damage and loss of the absorptive and digestive enterocytes (ECs), the predominant cell type in the gut epithelium^14^. In order to compensate for the loss of ECs, the gut possesses protective homeostatic mechanisms relying on the activity of intestinal stem cells (ISCs) that are scattered along the midgut epithelium^14^,^15^,^16^. Upon damage, the *Drosophila* midgut initiates a homeostatic feedback loop that couples EC loss to ISC division and differentiation. Several signaling pathways that are involved in *Drosophila* gut regeneration have been identified, such as the Janus kinase/signal transducer and activator of transcription (JAK/STAT) and the epidermal growth factor receptor (EGFR) pathways^14^,^17^,^18^,^19^,^20^. Gut damage induces the production of secreted ligands of Unpaired (Upd1, Upd2 and Upd3) and EGF (Spitz, Vein, Keren) families, which activate respectively the JAK/STAT and EGFR pathways in ISCs to promote their rapid proliferation and differentiation, thereby establishing homeostatic regulatory loops^21^,^22^.

The feeding habits of *Drosophila* species do not allow these flies to transmit diseases to humans. Mosquitoes by contrast transmit diseases through pathogen-infected meals. This makes the study of mosquitoes’ gut physiology and immunity highly relevant to human health. Some previous reports suggested the existence of ISCs in mosquitoes’ gut based on morphological characteristics^23^,^24^,^25^,^26^. Among these, in 1953 Day and Bennetts studied wound healing in the guts of *Aedes aegypti;* and in 1977, Hook evoked the presence of proliferating cells in *Culex tarsalis* guts after blood meal. In this study, we show that the gut of adult *A. albopictus* mosquitoes contains mitotic cells, which become highly proliferative upon ingestion of damaging chemical compounds or enteric bacterial infections. We also provide insight into the molecular pathways activated in the mosquitoes’ gut after damage.

## Results

### Structure of the adult *Aedes albopictus* gut

**Figure 1:**
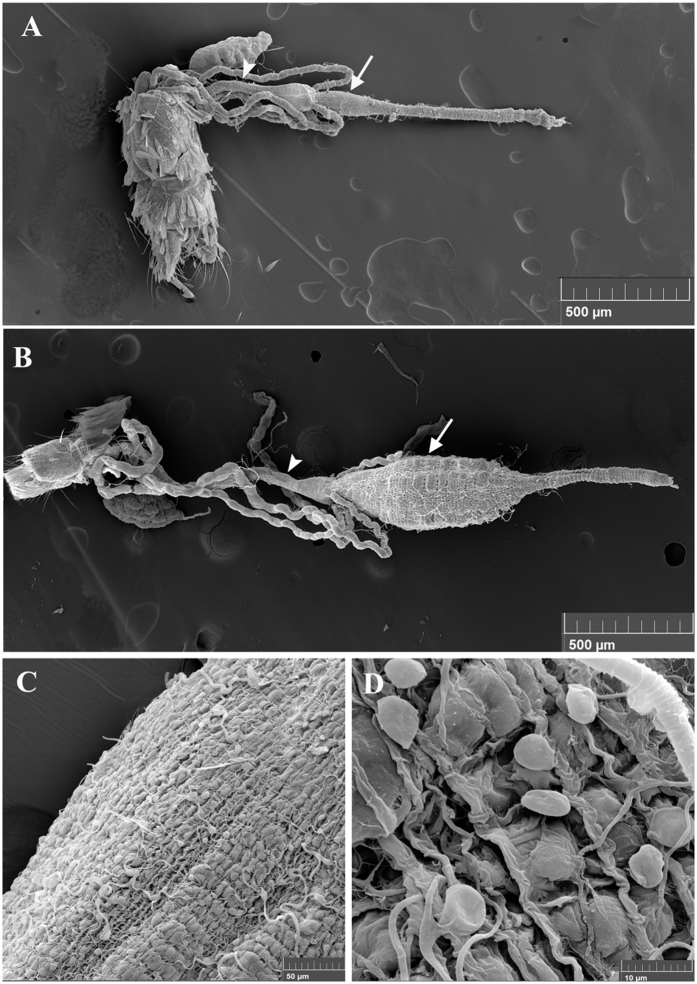
Scanning Electron Microscopy (SEM) images of *A. albopictus* mosquito guts. The ultrastructure of the gut of a male (**A**) and a female (**B**) as revealed by SEM shows two of the three main compartments: the hindgut (arrowhead) with the associated malpighian tubules and the midgut (arrow). (**C** and **D**) are close-up photos of female guts where individual visceral muscle cells as well as tracheal cells and tubing surrounding the gut are visible.

The adult mosquito gut consists of a simple epithelial tube divided into three discrete structures: the foregut, the midgut, and the hindgut. The foregut allows sugar solutions intake by the mouth, unidirectional passage of digesta through the pharynx and the esophagus and its storage in the crop, which is a bag like structure^27^. The midgut serves in food digestion and nutrients absorption and the hindgut with its associated malpighian tubules (functional analogues to mammalian kidney) plays excretory and osmoregulatory roles^28^. The midgut and hindgut are revealed in Fig. 1 using scanning electron microscope (the foregut is not shown). In addition, this figure shows a clear anatomical difference between male and female guts, male guts being overall smaller. In particular, the midgut compartment is less developed in males as compared to females (Fig. 1A and B). This is in agreement with the fact that female mosquitoes require a protein-rich blood meal to produce eggs, while males only consume sugars and are smaller than females. Therefore, male guts have to perform simpler digestive functions. The gut is surrounded by visceral muscles and a dense network of tracheal tubes delivering oxygen to and removing carbon dioxide from the gut cells (Fig. 1C and D).

### Establishment of a model to induce damage to the gut

**Figure 2:**
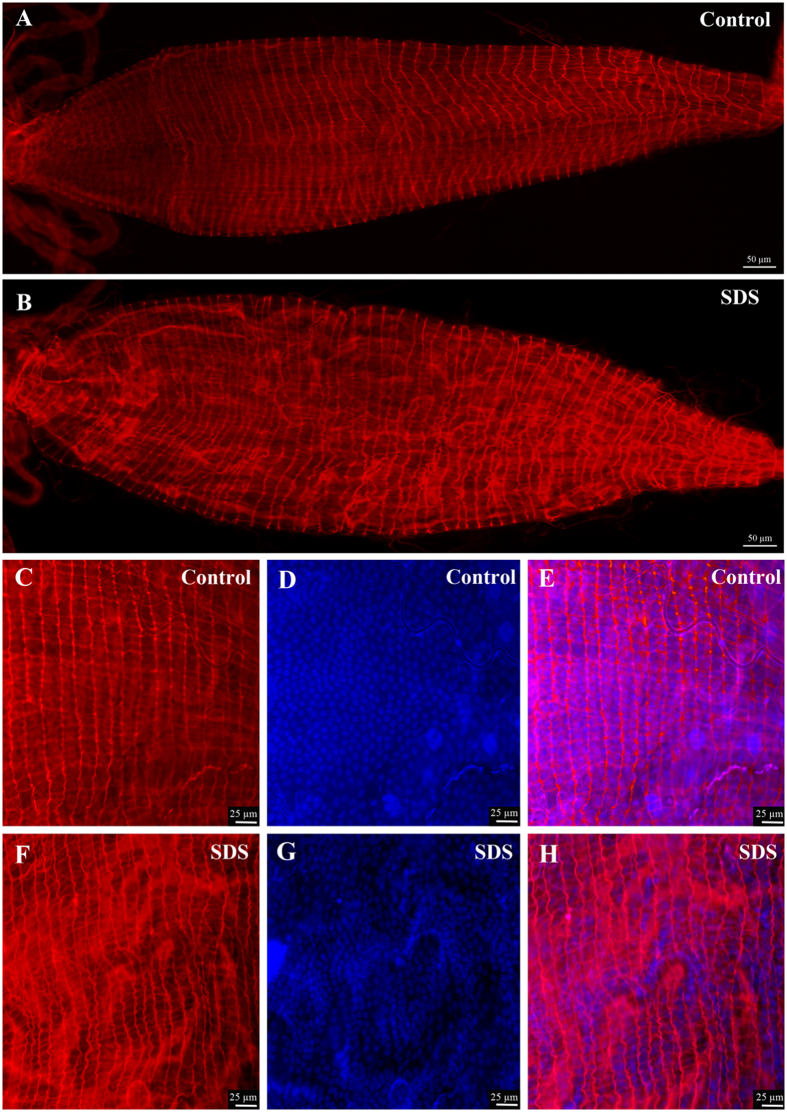
SDS feeding induces damage to the gut. Phalloidin and DAPI staining of dissected guts shows that the guts of mosquitoes fed for 24 hours on sucrose supplemented with 2% SDS have an altered structure (**B**) as compared to controls fed on sucrose (**A**). This can be better appreciated in higher magnification captures showing Phalloidin staining (**C** and **F**), DAPI staining (**D** and **G**) and the merged pictures (**E** and **H**).

To date, it is not known how the mosquito gut integrity is maintained and regulated when enterocytes suffer damage. To induce damage to mosquito guts, insects were starved for two hours then fed on a 10% sucrose solution containing 2% Sodium Dodecyl Sulfate (SDS), a chemical used in previous studies to induce damage in *Drosophila* guts^29^. Mosquito guts were dissected 24 hours post treatment and fixed. Staining with fluorescent phalloidin reagent that label the F-actin filaments allowed us to visualize the global morphology of the gut at different focal planes in order to compare the guts of SDS treated animals to those of the control group. As shown in Fig. 2, the guts of mosquitoes fed on sucrose supplemented with SDS (Fig. 2B,F,G and H) were distorted and the F-actin filaments did not show the same homogeneity when compared to the gut of control mosquitoes (Fig. 2A,C,D and E).This indicates that SDS treatment is a convenient and reproducible method to inflict damage to mosquito guts and therefore constitute a good model to analyze the effect of damage on gut structure and physiology.

### Proliferating cells are present in the guts of *Aedes albopictus* mosquitoes

**Figure 3:**
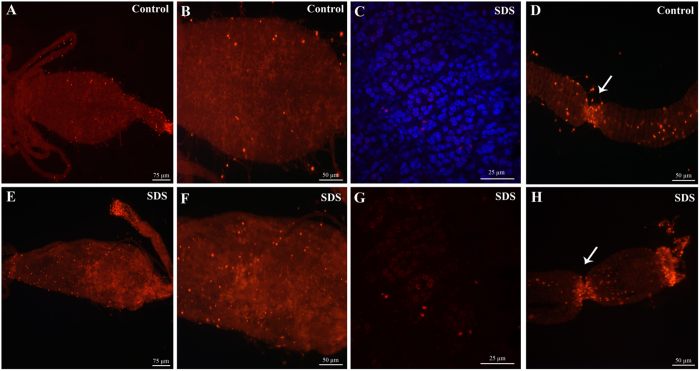
Regenerative cells are present in the midgut of adult *A. albopictus*. Immunofluorescence staining using anti-PH3 antibodies reveals the presence of cells undergoing division in the gut of adult mosquitoes. An increase in the number of proliferating cells in the midgut is observed 24 hours after feeding the mosquitoes on SDS-sucrose (**E** and **F**) as compared to the midguts of control mosquitoes (**A** and **B**). A higher magnification picture focused on midgut cells labeled with anti-PH3 and DAPI staining is shown in (**C** and **G**). Two zones of active cell division (arrows) are observed in the most anterior part of the midgut independently of gut damage (**D** and **H**).

To unravel the existence of proliferating cells in the gut of mosquitoes, we stained the tissue with antibodies raised against phospho-histone H3 protein (anti-PH3), a specific marker of cells undergoing mitosis^30^,^31^. Staining was performed on guts of mosquitoes fed either on sucrose or SDS. Low numbers of small cells with strong PH3 labeling were observed in the midguts of control mosquitoes (Fig. 3A and B). In many cases, two PH3 positive nuclei were observed close to each other, these had a characteristic coffee-bean shape and very likely correspond to two sister cells originating from the recent division of a progenitor cell. A number of PH3 positive cells were also observed in the tracheal tubes surrounding the midgut, these can be easily distinguished from small PH3 positive cells located inside the gut epithelium based on their bigger sized nuclei and on their location and arrangement. A higher magnification picture focused on midgut cells labeled with anti-PH3 and DAPI staining is shown in Fig. 3C and G. When compared to the guts of control mosquitoes, the guts of SDS fed mosquitoes appeared damaged and distorted confirming the observation shown in Fig. 2. Interestingly, an increase in the number of PH3 positive cells was observed (Fig. 3E and F) in comparison to control guts. These findings suggest that SDS feeding induces gut damage resulting in the activation of local regenerative responses. In addition, we were able to detect both in control guts (Fig. 3D) and in damaged guts (Fig. 3H) the presence of a high numbers of dividing cells in a restricted area of the most anterior part of the midgut.

### Chemical and bacterial damage induce a significant increase in the number of mitotic cells

**Figure 4:**
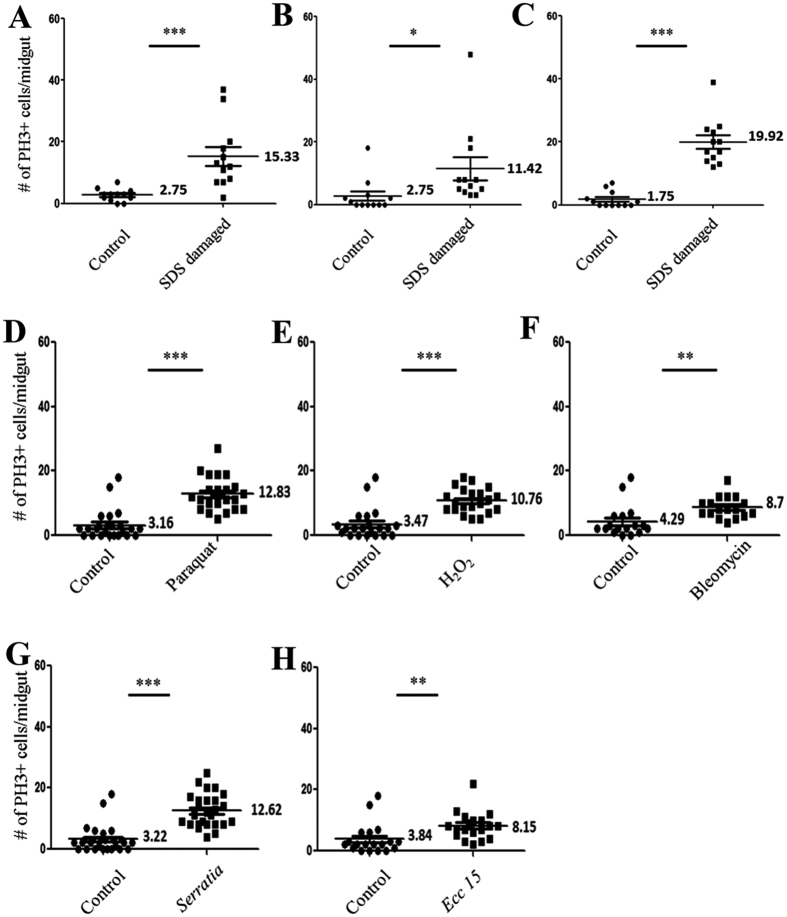
Feeding on stress inducing chemicals or on pathogenic bacteria increases cell division in the midgut. The number of dividing cells stained by anti-PH3 antibodies per midgut is counted and statistical analysis confirms a significant difference in the number of proliferating cells in damaged guts as compared to control ones. For SDS feeding the experiment was done in triplicates (**A**–**C**), and the number of guts analyzed is n = 12 for each replicate. For paraquat feeding (**D**) n = 24, H_2_O_2_ (**E**) n = 21, Bleomycin (**F**) n = 17. For *Serratia marcescens* (**G**) n = 26 and *Erwinia carotovora carotovora 15* (**H**) n = 19. ***P < 0.001, **P < 0.01, *P < 0.05.

First, we performed a quantification of PH3 positive cells residing in the midgut epithelium of mosquitoes fed on sucrose or SDS solutions. Three independent experiments were done with 12 guts analyzed for each condition per experiment. We also used other chemicals that are classically used to induce gut damage in *Drosophila melanogaster,* including paraquat, H_2_O_2_ and Bleomycin^20^,^29^. Cell counts were plotted using the GraphPad Prism software and results are shown in Fig. 4A–F. For all experiments, a statistically significant difference was found between the numbers of PH3 positive cells in damaged versus control guts. The average number of dividing cells in control guts was 2.42 versus 15.56 in SDS treated guts (n = 36). For paraquat (n = 24), H_2_O_2_ (n = 21) and for Bleomycin (n = 17) feeding, the average number of dividing cells per gut as compared to control was respectively: 12.83 versus 3.16; 10.76 versus 3.47 and 8.70 versus 4.29.

To investigate whether enteric infections can also trigger cell proliferation in the mosquito midgut, we used the pathogenic Gram-negative bacteria *Serratia marcescens* and *Erwinia carotovora carotovora 15 (Ecc15*). We fed *Aedes* mosquitoes on a sucrose solution containing a high concentration of each bacterial suspension (OD 50). In both cases, we observed a significant increase in the number of dividing cells per midgut: 12.62 for *S. marcescens* as compared to 3.22 for control guts (n = 26) and 8.15 for *Ecc15* as compared to 3.84 for controls (n = 19) (Fig. 4G,H and Supplementary Figure 1). These results suggest that damaging the gut of mosquitoes triggers an intrinsic increase in cell proliferation.

### Ingestion of pathogenic bacteria induces local AMP production in the gut

**Figure 5:**
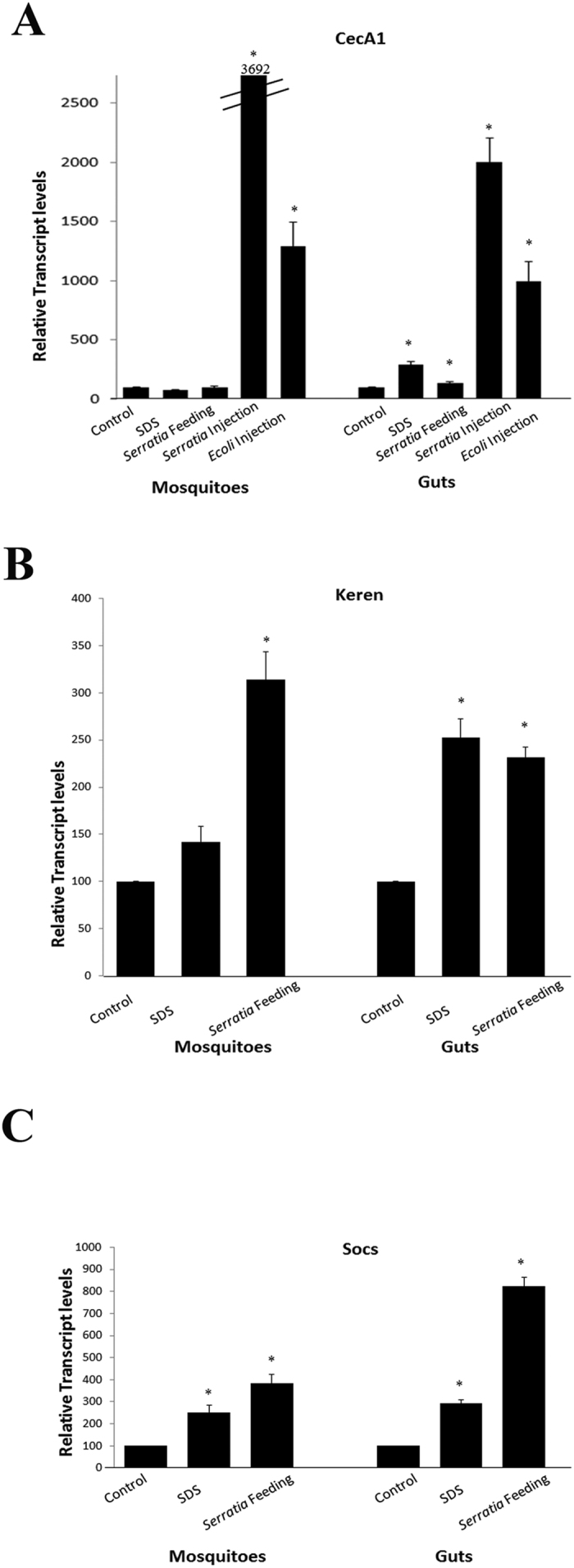
Transcriptional levels of genes encoding CecropinA1 AMP and signaling molecules belonging to the EGFR and JAK/STAT pathways after gut damage. Feeding *Aedes* mosquitoes on sucrose containing 2% SDS or a high concentration of *Serratia marcescens* (OD = 50) leads to a slight increase in the transcription of the antimicrobial peptide CecA1 encoding gene in the gut. However, this increase was not observed in whole insects, where the levels of AMP transcripts are highly induced only when bacteria (*Serratia* or *E. coli*) is microinjected into the mosquito body cavity causing a systemic immune response (**A**). Real time qPCR performed on whole mosquitoes or on dissected guts shows that feeding on *Serratia* results in a significantly increased transcription, both at the level of the gut and in whole animals, of the signaling molecules *Keren* (**B**) and *Socs* (**C**) that are known to regulate in *Drosophila* the EGFR and JAK/STAT pathways respectively. SDS feeding also led to a significant increase of *Keren* and *Socs* at the level of the gut (**B** and **C**). *P < 0.05.

We assessed the expression levels of the antimicrobial peptide CecropinA1 (CecA1) gene in *S. marcescens* fed mosquitoes using qRT-PCR. This AMP is believed to be active against Gram-negative bacteria based on work realized in *Drosophila melanogaster*^32^,^33^. CecA1 transcripts levels were slightly but significantly increased (by approximately 1.4 fold) in the guts of orally infected mosquitoes as compared to controls (Fig. 5A). CecA1 transcriptional upregulation was not observed in whole mosquitoes orally infected with *S. marcescens*, indicating that only a local response was triggered in the gut. This result is in agreement with what has been observed in *Drosophila* after *S. marcescens* feeding^34^; it also gives additional molecular evidence that the mosquito gut acts as a first barrier to protect the organism from foreign invaders. For comparison, we injected *S. marcescens* and *Escherichia coli* bacteria into the body cavity of mosquitoes to induce the systemic immune response and observed very high expression levels of CecA1 (respectively 360 and 120 folds as compared to non-injected controls) (Fig. 5A).

### Analysis of candidate signaling pathways genes expression after gut damage

To gain insight into the molecular signaling pathways activated in damaged mosquito guts, we looked in the recently released genome of *A. albopictus* for genes with orthologous counterparts that were known to be involved in gut regeneration in *Drosophila*. We were not able to identify an orthologue to any of the three *Drosophila unpaired* genes (JAK/STAT ligands) in *A. albopitcus* genome. However, we found an orthologous of the *Socs36E*, the known target and negative regulator of the JAK/STAT pathway in *Drosophila*^35^,^36^. Alternatively, we looked for the ligands of the EGFR pathway that are highly induced in the *Drosophila* midgut following stress-induced damages^37^. The ligands are usually more up- or down-regulated as compared to receptors or intracellular components of signaling pathways. We identified an *A. albopictus* orthologue to the *Drosophila Keren* gene, one of the four known EGFR ligands in *Drosophila*.

Using specific primers that amplify those genes, we performed qRT-PCR on whole mosquitoes fed with SDS, with *S. marcescens* or with sucrose. We also did qRT-PCR on guts isolated from mosquitoes that received these three different treatments. SDS feeding led to a significant increase of both *keren* (2.5 folds) and *Socs36E* (2.9 folds) transcripts in the guts (Fig. 5B and C). This figure shows also a significant increase in the transcripts levels, both in dissected guts (2.3 folds for *keren* and 8.2 folds for *Socs36E*) and in whole animals (3.1 folds for *keren* and 3.8 folds for *Socs36E*), after *S. marcescens* feeding. These results suggest that EGFR and JAK/STAT pathways may be involved in the regenerative response triggered in the adult *A. albopictus* gut following local cell damages.

## Discussion

Mosquitoes are one of the deadliest insects responsible of the transmission of diseases that have dramatic impact on human health. In total, vector-borne diseases account for approximately 17% of the estimated burden of all infectious diseases^38^. Pathogens such as bacteria, viruses and parasites complete part of their life cycle in the insect midgut, where they are confronted to mosquito-encoded barriers and host effectors that can restrict their development. Therefore, the characterization of the cellular and molecular mechanisms required to maintain normal mosquitoes gut structure and function is highly demanded and could provide novel control strategies of diseases vectors.

In this study, we have investigated the ability of the adult *A. albopictus* gut to regenerate in response to chemical or bacterial challenges. We were able to show the existence of small proliferative cells in the midgut of *A. albopictus*, which are probably intestinal stem cells. These cells showed a regenerative behavior in response to local gut damages induced either by chemical compounds or by enteric bacterial infections. At the molecular level, the gene expression of known components of the JAK/STAT and EGFR pathways were significantly induced in response to gut damages. We did also observe a clear difference in gut size and proportions (especially in the midgut) between males and females, but this is not surprising since males and females have different feeding habits.

SDS feeding triggered a moderate upregulation of the genes encoding the signaling molecules Keren (approximately 2.5 folds) and Socs36E (approximately 2.9 folds) in the gut of *A. albopictus*. These results indicate that signaling via EGFR and JAK/STAT pathways is due to gut damage and the possible entry of bacteria from the intestinal lumen to the body cavity. This signaling is enhanced by the presence of pathogenic bacteria and the immune response it triggers. Indeed, *Serratia* feeding causes a higher activation of transcription of *Socs36E* and of *Keren*.

Our findings are in agreement with what has been observed in *Drosophila melanogaster*, despite some striking differences between *A. albopictus* and *D. melanogaster* genomes: the DNA content of *Aedes* genome is more than 10 times that of *Drosophila*^39^. Noteworthy, no orthologous counterpart for several *Drosophila* key immunity genes (such as the *upd* genes) were identified in *A. albopictus*, possibly due to gaps in the first release of the genome. The identification of more orthologues to *Drosophila* genes that participate in pathways controlling intestinal cell division and differentiation is crucial in order to characterize the gut regenerative response in *A. albopictus.* This would help also to describe in an exhaustive manner the intestinal stem lineage in *A. albopictus*. On another hand, a reverse genetics approach should be followed to achieve candidate gene knock-down and determine the contribution of each pathway to the regulation of intestinal stem cell activity.

Extensive published findings from the *Drosophila* model will certainly help to a better characterization of the mechanisms underlying mosquitoes gut homeostasis^40^. Our study provides evidence for the existence of regenerative cells and shows that they proliferate in damaged *Aedes* guts. These results should contribute to a better understanding of how the gut homeostasis is maintained and, together with a more in depth characterization of mosquito’s immune responses, should pave the way for a the development of alternative control strategies of theses disease vectors.

## Material and Methods

### Mosquito rearing

All animal procedures were carried according to protocols approved by the Institutional Animal Care and Use Committee (IACUC) at the American University of Beirut, and all methods were carried out in accordance with relevant IACUC guidelines and regulations.

A local strain of *Aedes albopictus* mosquitoes (originally captured from Sarba in the suburbs of Beirut, Lebanon) was maintained in the insectary at 28 °C and 75% humidity using a 12:12 light:dark photocycle. Adults were continuously supplied with cotton pads soaked in a 10% sucrose solution and had access to water cups containing clean tap water. Feeding was allowed on anesthetized mice and eggs were collected on filter paper four days after the blood meal. Eggs were dried for two weeks before hatching was attempted by immersion in aged tap water. After hatching, larvae were fed on yeast for the first 24 hours then on fish pellet food till pupation. Pupae were collected with a plastic pipette and placed in water cups inside plastic cages.

### Chemical and bacterial treatments

Mosquitoes were starved for 2 hours before their cups were supplemented with cotton pads soaked in 10% sucrose (for controls), 2% SDS −10% sucrose, or 0.3% H_2_O_2_ −10% sucrose, or 4 mM Paraquat (Sigma-Aldrich, USA) −10% sucrose, or 25 μg/ml Bleomycin (Cell Pharm, Germany) −10% sucrose (for the induction of chemical damage) or a bacterial suspension (OD = 50) in 10% sucrose (for infection experiments). The mosquitoes were allowed to feed continuously until the guts were dissected (24 hours after the treatment for immunohistochemistry or 12 hours for real time PCR analysis). The bacterial strains used in this experiment were *Serratia marcescens* pGEN222 and *Erwinia carotovora carotovora 15 (Ecc15*).

### Isolation of mosquito midguts

Mosquitoes were cold anesthetized by placing the cups on ice, and transferred one at a time onto a glass slide in a drop of 1X PBS. Isolation of midguts was performed under a light stereomicroscope. Using fine forceps, the animal head was cut and the mosquito abdomen was pulled from the posterior end until the midgut detaches. The isolated midguts were then placed in 1.5 ml eppendorf tube containing 1X PBS and kept on ice.

### Fixation and staining

Isolated guts were fixed for 30 minutes using a 4% Parafolmadehyde (VWR, USA) solution in 1X PBS. This was followed by three 15-minute washes in PBS-Triton 0.1% to allow permeabilization of the guts. Blocking was then performed for 30 minutes by adding a solution of 1X PBS -Triton 0.1%-BSA 1%. After blocking, the primary rabbit α-PH3 antibodies (ABCAM, UK) were added (1:800 in 1X PBS-Triton 0.1%-BSA 1%) overnight at 4 °C. Following three 15 minute washes in PBS-Triton 0.1%, the secondary antibodies Alexa Fluor^®^ 555 (ABCAM, UK) were added (1:1000 in PBS-Triton 0.1%-BSA 1%) for three hours at room temperature. Phalloidin coupled to Alexa Fluor^®^ 647 (ABCAM, UK), and was added for one hour at room temperature (1:500 in PBS-Triton 0.1%-BSA 1%). After secondary antibodies or phalloidin removal, DAPI stain was applied at a concentration of 1:10 000 for 2 minutes, then three final washes in PBS-Triton 0.1% were performed, guts were mounted on microscope slides in anti-fade medium (Immu-Mount, Thermo Scientific) and coverslips were sealed with colorless nail varnish.

### Microscopy, cell counting and statistical analysis

The slides prepared were observed under an inverted fluorescence microscope (Zeiss Axiovert 200, Source: AttoArc2 HBO 100 W) for the counting of proliferating cells and an upright fluorescence microscope (Leica DM6 B) for image acquisition using the image stitching option. Cell counts were analyzed using the Graphpad Prism software and an unpaired t test was performed.

### Scanning electron microscopy

Midguts were dissected and incubated for two hours at room temperature using a PBS fixative solution containing 25% glutaraldehyde and 4% parafolmadehyde. After three 5 minute washes in 1X PBS, the guts were dehydrated using increasing concentrations of ethanol in the following steps: 2 hours in 30% ethanol, overnight in 50% ethanol, 6 hours in 70% ethanol and finally overnight in 100% ethanol. The guts were then dried in a critical point dryer (EMS Quorum 850), coated in gold and observed under the MIRA3 LM TESCAN scanning electron microscope (SEM High Voltage: 15 kV, Detector Oxford Instruments X-Max: SE).

### Bacteria microinjection

Thirty two nanoliters of an *Escherichia coli* (DH5 alpha laboratory strain) bacterial suspension of optical density (OD) = 0.15 or a *Serratia marcescens* pGEN222 bacterial suspension of optical density (OD) = 0.01 were injected into the thorax of insects using a Nanoject II apparatus (Drummond Scientific, CA). Each experiment was performed using a minimum of 15 mosquitoes.

### Real-time PCR

Whole mosquitoes or dissected guts were directly placed and homogenized in TRIzol^®^. RNA was extracted using choloroform and precipitated with isopropanol according to the manufacturer’s instructions (Invitrogen). The extracted RNAs were quantified using a nanodrop spectrophotometer (Thermo) and 500 ng were retrotranscribed into cDNA (iScript Biorad) for each sample. Real-time PCR was performed in presence of SYBR green (Qiagen) on 1/20 dilutions of the RT reactions using a BIO RAD thermocycler (CFX 96 Real-time System, C1000). Ct values for target genes were normalized to Rp49 and compared to controls using the delta Ct method. A minimum of three independent experiments were averaged and unpaired t tests were performed. Primers were designed using Primer3 online software, except for CecA1 they were copied from^41^. Primers used were:

Rp49 Forward: 5′-AGTCGGACCGCTATGACAAG-3′

Rp49 Reverse: 5′-GACGTTGTGGACCAGGAACT-3′

CecA1 Forward: 5′-GAGTCGGCAAACGAGTCTTC-3′

CecA1 Reverse 5′-TTGAACCCGGACCATAAATC-3′

Socs Forward 5′-TCGACTTCATCCACTGCTTG-3′

Socs Reverse: 5′-ACGACACGGAAAACAGGAAC-3′

Keren Forward: 5′-TGATGATCCATTTCGCAAGA-3

Keren Reverse: 5′-CTTATCCGTCTCCTGCCTGA-3′.

## Additional Information

**How to cite this article:** Janeh, M. *et al*. Damage-Induced Cell Regeneration in the Midgut of *Aedes albopictus* Mosquitoes. *Sci. Rep.* **7**, 44594; doi: 10.1038/srep44594 (2017).

**Publisher's note:** Springer Nature remains neutral with regard to jurisdictional claims in published maps and institutional affiliations.

## Supplementary Material

Supplementary Figure 1
