# Supplementary material for: Damage-Induced Cell Regeneration in the Midgut of Aedes albopictus Mosquitoes
Source: Sci Rep. 2017 Mar 16;7:44594. doi: 10.1038/srep44594 (PMC5353711; doi:10.1038/srep44594)
Supplement: Supplementary Figure 1 [file srep44594-s1.pdf]

Damage-Induced Cell Regeneration in the Midgut of *Aedes albopictus* Mosquitoes

Maria Janeh[1], Dani Osman[2] and Zakaria Kambris[1*]

[1] Biology Department, Faculty of Arts and Sciences, American University of Beirut, Beirut, Lebanon
[2] Faculty of Sciences III and Azm Center for Research in Biotechnology and its Applications, EDST, Lebanese University, 1300, Tripoli, Lebanon
* for correspondence: zakaria.kambris@aub.edu.lb

Supplementary Figure 1:
Bacterial feeding increases the number of PH3 positive cells in the guts of adult *A. albopictus*

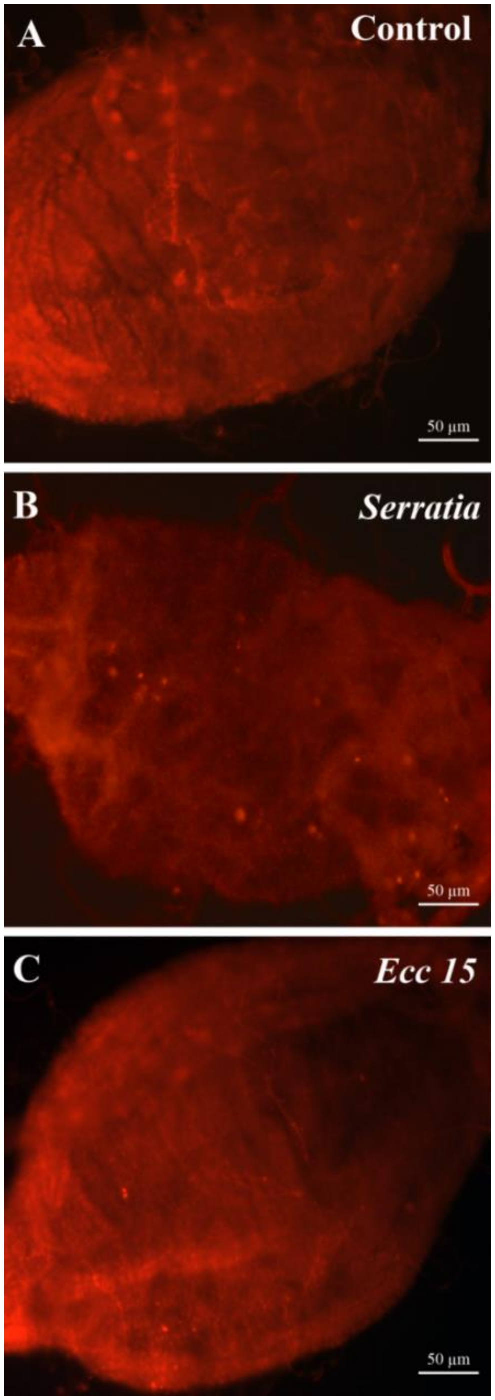

Supplementary Figure 1: Bacterial feeding increases the number of PH3 positive cells in the guts of adult *A. albopictus*

The number of proliferating cells in the midgut increases after feeding the mosquitoes on a sucrose solution containing a high concentration of *S. marcescens* (B) or *Ecc15* (C) for 24 h as compared to the midguts of control mosquitoes (A) (The figures shown are representative of 26 guts analyzed for *S. marcescens* and 19 guts for *Ecc15*; the complete statistics are shown in Figure 4).
